# Supplementary figures and images for: Resting-State EEG Functional Connectivity in Children with Rolandic Spikes with or without Clinical Seizures
Source: Biomedicines. 2022 Jun 29;10(7):1553. doi: 10.3390/biomedicines10071553 (PMC9312817; doi:10.3390/biomedicines10071553)

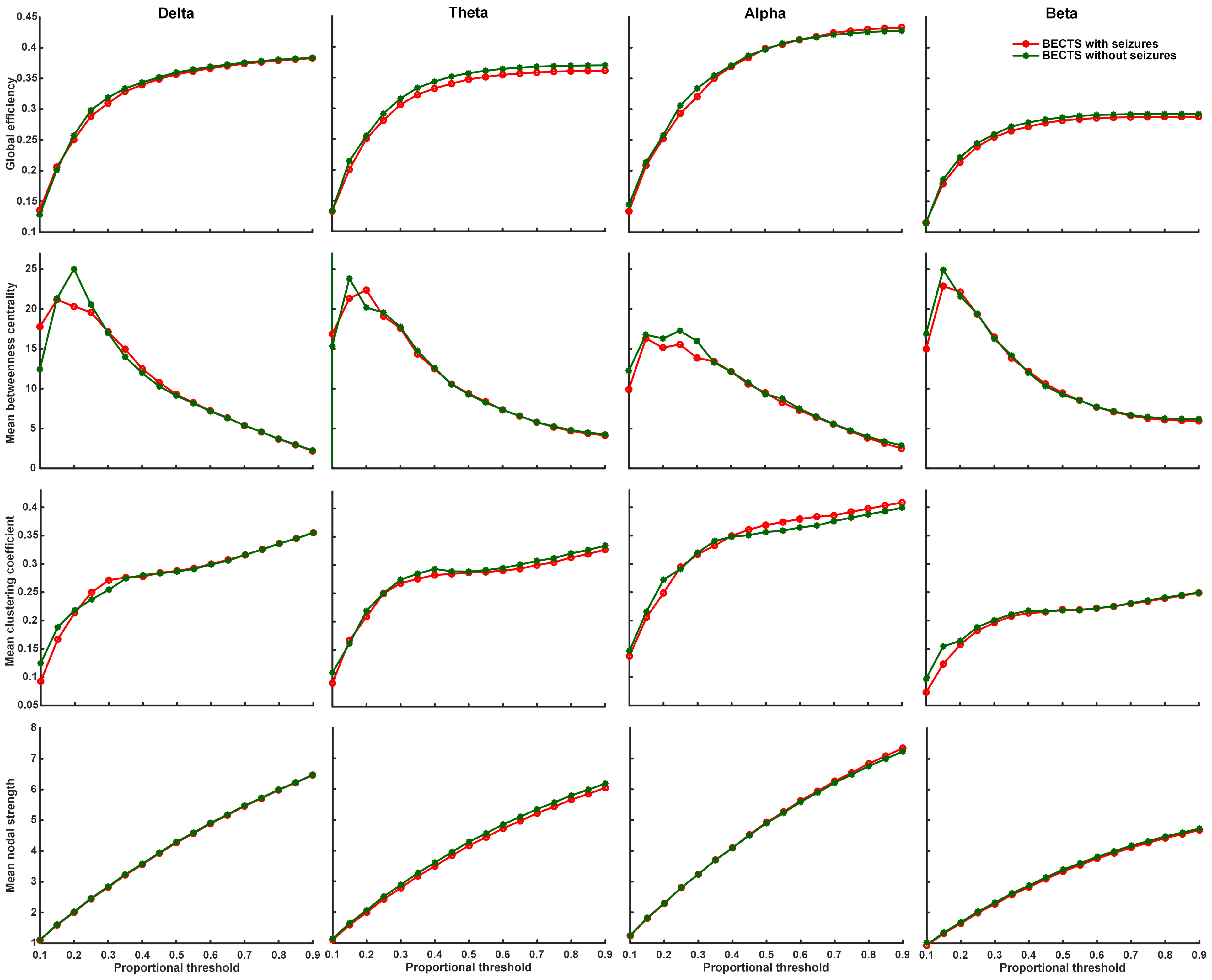

Supplement: Supplementary file 1 [file biomedicines-10-01553-s001.zip › Figure S1.pdf]
